# Neutrophil gelatinase-associated lipocalin as a marker of tubular damage appears to be unrelated to fractional excretion of sodium as a marker of tubular function in septic patients, with or without AKI

**DOI:** 10.1186/cc10379

**Published:** 2011-10-27

**Authors:** NJ Glassford, AG Schneider, G Eastwood, L Peck, H Young, R Bellomo

**Affiliations:** 1 Department of Intensive Care, Austin Hospital, Heidelberg, VIC, Australia

## Introduction

The utility of urinary biochemistry has recently been challenged [1], while there is emerging evidence that renal biomarkers may accurately quantify the risk of development of acute kidney injury (AKI) [2]. Neutrophil gelatinase-associated lipocalin (NGAL) is a marker of renal tubular damage [3]. Fractional excretion of sodium (FENa) is a marker of renal tubular function, and is a significantly cheaper investigation [4]. Insults damaging the tubules and resulting in AKI should both stimulate NGAL production and prevent resorption of sodium. Given the different pathological mechanisms underlying septic and nonseptic AKI, it is plausible that the relationship between these variables could be different in these two groups of patients [5].

## Methods

To test this hypothesis, we studied ICU patients developing SIRS and oliguria or a 25 μmol/l increase in serum creatinine within 48 hours of ICU admission. We sought to determine if a relationship existed between FENa and NGAL in patients, with and without sepsis, developing AKI. We measured the serum and urinary NGAL, creatinine and sodium of patients with SIRS and either oliguria or an increase in creatinine within 48 hours of admission to a tertiary referral ICU. Point-of-care creatinine measurements were used to identify the maximum RIFLE category of AKI developed within the first 5 days of admission. The strength of the relationship between variables was determined using Spearman's rank correlation coefficient.

## Results

We enrolled 93 patients between 31 August 2010 and 17 November 2010; 17 had an APACHE III diagnosis of sepsis. Serum NGAL and urinary NGAL when corrected for urinary creatinine were found to correlate moderately well with FENa in patients without sepsis, a relationship that weakens with the progression of AKI in this group. No other correlation showed a significant relationship (Table 1).

**Table 1:** Relationships between NGAL, FENa and AKI

			Urinary NGAL		Urinary NGAL corrected for urinary creatinine		Serum NGAL		Urine:serum NGAL ratio	
						
FENa		*n*	Spearman	*P *value	Spearman	*P *value	Spearman	*P *value	Spearman	*P *value
Nonseptic	No AKI	43	0.153	0.328	0.587	<0.0001	0.450	0.002	-0.009	0.953
	AKI	33	-0.039	0.830	0.438	0.011	0.258	0.148	-0.235	0.188
	RIFLE R-F	14	0.015	0.958	0.235	0.418	-0.077	0.793	-0.068	0.817
Septic	No AKI	12	-0.168	0.602	-0.007	0.983	0.232	0.467	-0.427	0.167
	RIFLE R-F	5	-0.300	0.624	0.500	0.391	0.112	0.858	-0.400	0.505

## Conclusion

The lack of a strong correlation FENa and NGAL in patients developing RIFLE I and F AKI suggests that changes in NGAL and changes in sodium resorption occur as a consequence of different stimuli in the pathogenesis of the syndrome. The absence of any observed relationship between NGAL and FENa in septic patients suggests a pathological process different from that underlying nonseptic AKI. The small sample size may be a confounding factor.

## References

[B1] Bagshaw SM Langenberg C Wan L May CN Bellomo R A systematic review of urinary findings in experimental septic acute renal failure Crit Care Med 2007 35 1592 1598 10.1097/01.CCM.0000266684.17500.2F 17452939

[B2] Siew ED Ware LB Ikizler TA Biological markers of acute kidney injury J Am Soc Nephrol 2011 22 810 820 10.1681/ASN.2010080796 21493774

[B3] Cruz DN de Cal M Garzotto F Perazella MA Lentini P Corradi V Piccinni P Ronco C Plasma neutrophil gelatinase-associated lipocalin is an early biomarker for acute kidney injury in an adult ICU population Intensive Care Med 2010 36 444 451 10.1007/s00134-009-1711-1 19956925 PMC2820221

[B4] Kanbay M Kasapoglu B Perazella MA Acute tubular necrosis and pre-renal acute kidney injury: utility of urine microscopy in their evaluation - a systematic review Int Urol Nephrol 2010 42 425 433 10.1007/s11255-009-9673-3 19921458

[B5] Ishikawa K May CN Gobe G Langenberg C Bellomo R Pathophysiology of septic acute kidney injury: a different view of tubular injury Contrib Nephrol 2010 165 18 27 20427951 10.1159/000313740

